# Amphibians and reptiles of C. E. Miller Ranch and the Sierra Vieja, Chihuahuan Desert, Texas, USA

**DOI:** 10.3897/zookeys.735.22200

**Published:** 2018-02-06

**Authors:** Drew R. Davis, Travis J. LaDuc

**Affiliations:** 1 Department of Biology, University of South Dakota, 414 East Clark Street, Vermillion, South Dakota 57069, USA; 2 Biodiversity Collections, The University of Texas at Austin, 10100 Burnet Road, PRC 176–R4000, Austin, Texas 78758, USA

**Keywords:** Amphibia, Checklist, Herpetological diversity, Land stewardship, Long-term survey, Reptilia, Species composition, Voucher collections

## Abstract

We report the occurrence of 50 species of amphibians and reptiles recently collected on C. E. Miller Ranch and the Sierra Vieja in the Chihuahuan Desert of Texas, USA and describe their perceived distribution and abundance across various habitat associations of the region. Our recent surveys follow intense, historic sampling of amphibians and reptiles from this region in 1948. Of the 50 species detected in recent surveys, six were not collected in 1948 and an additional three species documented in 1948 have yet to be detected in a 14-year period of recent surveys. Combining data from both historic and recent surveys, a total of 53 species of amphibians and reptiles are known from the ranch (11 amphibians, 42 reptiles). Land stewardship and conservation practices have likely contributed to the persistence of the majority of these species through time. Additionally, we discuss the status of amphibians and reptiles not collected during recent surveys and comment on potential species that have not yet been detected.

## Introduction

The Chihuahuan Desert is a large arid region in North America that is home to high levels of biodiversity and is considered among the world’s most valuable ecoregions (Olson and Dinerstein 1998). This ecoregion occupies most of north-central Mexico where it is positioned on the Mexican Plateau and bounded on the east by the Sierra Madre Oriental and on the west by the Sierra Madre Occidental (Schmidt 1979). In the United States, the Chihuahuan Desert extends into southeastern Arizona, southern New Mexico, and most of the Trans-Pecos region of Texas (Ricketts et al. 1999).

Situated in the Chihuahuan Desert, the Sierra Vieja (also referred to as the Tierra Vieja, Vieja Mountains: Gannett 1904; Sierra Vieja Range: Jameson and Flury 1949, Philips and Thornton 1949; Sierra Tierra Viejas: York 1949) is a low-elevation mountain range (1500–1900 m) located north and east of the Rio Grande in western Jeff Davis and Presidio counties, Texas, USA that is approximately 65 km in length and primarily composed of igneous rock (York 1949). The Sierra Vieja serve as an important barrier between the Rio Grande Plain to the west and the Valentine Plain to the east, with its steep, incised canyons providing protection and habitat in an otherwise open terrain. Outside of Blair (1940), Schmidt and Smith (1944), and Schmidt and Owens (1944), little was known about the vertebrates of this region until a large expedition led by W. F. Blair from the University of Texas at Austin travelled to the Sierra Vieja to conduct biological surveys of plants and vertebrates from June–July 1948. This expedition collected a large number of specimens, all of which were deposited into the Texas Natural History Collections (now Biodiversity Collections; vertebrates) and the TEX Herbarium (now Billie L. Turner Plant Resources Center; plants) at The University of Texas at Austin and served as the foundation for four papers describing the flora and fauna of the area (mammals: Blair and Miller 1949; amphibians and reptiles: Jameson and Flury 1949; birds: Phillips and Thornton 1949; plants: York 1949). Over 1700 amphibian and reptile specimens were collected and vouchered during the 38-day survey, primarily by A. L. Carroll, T. M. Burke, D. L. Jameson, A. G. Flury, and W. F. Blair, with the assistance of 18 other undergraduate and graduate students who were part of the collecting party, providing a baseline sampling of species that occurred in this area in 1948. Additionally, at that time, many of these specimens were among the first to be collected for several species (i.e., *Bogertophis subocularis*, *Lampropeltis alterna*, *Trimorphodon vilkinsonii*; Jameson and Flury 1949).

Here we report the results from herpetological surveys conducted from 2004–2017 at the same locality that was sampled in 1948, C. E. Miller Ranch. We compare differences in species composition from 1948 to recent surveys, provide potential explanations for these patterns, and discuss future species that may yet be encountered. Additionally, we highlight the importance of land stewardship in maintaining amphibian and reptile diversity in the Chihuahuan Desert ecoregion.

## Methods

### Study site

**Figure 1.**
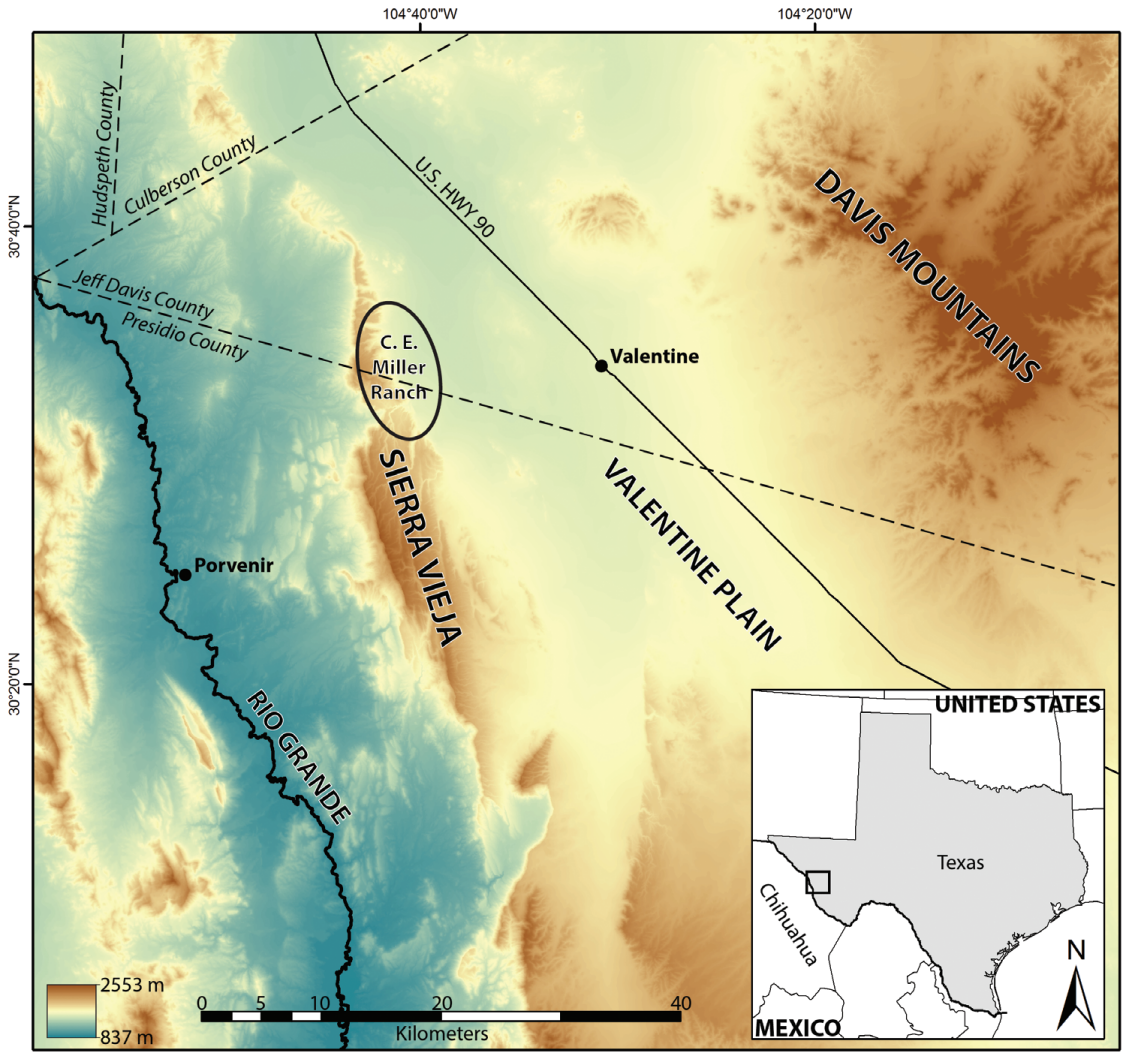
Map of C. E. Miller Ranch and the Sierra Vieja in the Chihuahuan Desert of Texas, USA. Major geographic features of the area, county boundaries, roads, and towns are included. Oval boundary around C. E. Miller Ranch is approximate.

**Figure 2.**
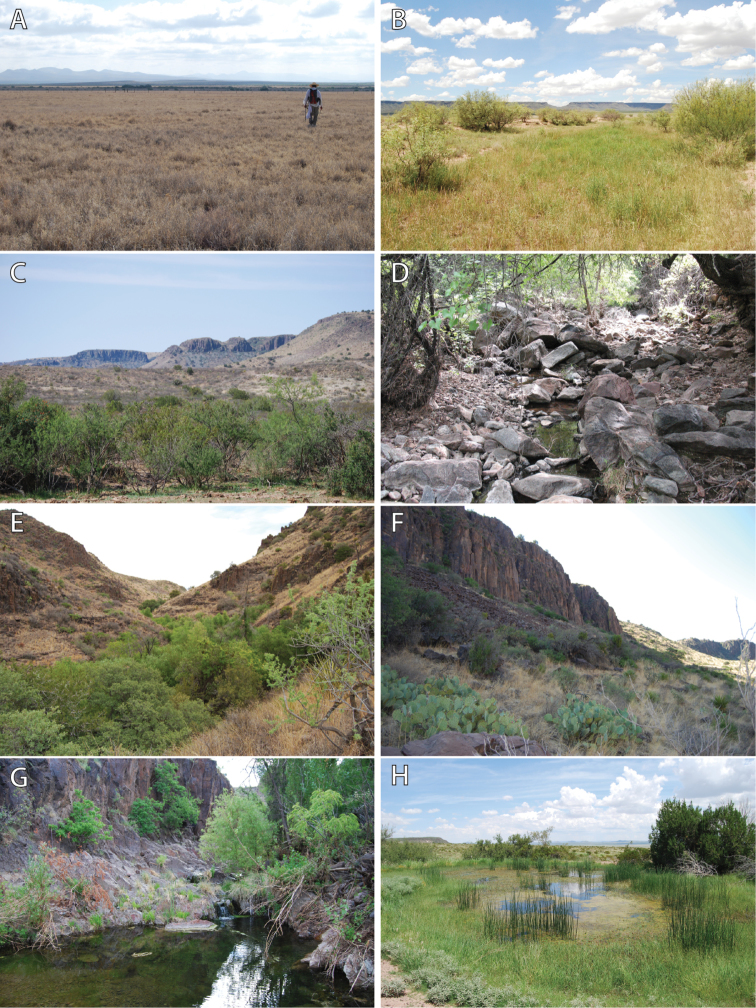
Photos of representative habitats present at C. E. Miller Ranch and the Sierra Vieja. **A** Valentine Plain, tobosa-grama association **B** Valentine Plain, mesquite-huisache-blackbrush association **C** Valentine Plain, creosote bush-catclaw-blackbrush association **D** Sierra Vieja, stream bed association **E** Sierra Vieja, catclaw-grama association **F** Sierra Vieja, grama-bluestem association **G** Sierra Vieja, Ojos Viejitas or Canyon Springs, and **H** Valentine Plain, 96 Tank. Photos by DRD.

C. E. Miller Ranch is located in western Jeff Davis and Presidio counties in west Texas and occurs in the Chihuahuan Desert ecoregion (Figure 1). This ranch is approximately 13,400 ha and has been a working cattle ranch for over 90 years. Additionally, the landowners have been awarded conservation and land stewardship awards for their efforts in maintaining wildlife habitat, promoting conservation, and partnering with various agencies and organizations (Klepper 2003). Much of the geology, climate, and vegetation of this region has previously been described by Baker (1927), Hinckley (1947), and York (1949). Large canyons in the Sierra Vieja empty out onto the Valentine Plain with wide alluvial fans occurring, suggesting a history of flash flooding and rock and sediment transport. Primary aquatic habitats on the ranch include a series of springs and pools that occur in canyons in the Sierra Vieja, temporary to semi-permanent pools in Wild Horse Draw on the Valentine Plain, and a series of semi-permanent and permanent dugout ponds that are water sources for livestock and wildlife (Fig. 2). Ojos Viejitas or Canyon Springs (Brune 1981; Fig. 2G), located near the eastern end of ZH Canyon, is one the most significant and isolated water sources available across the length of the Sierra Vieja, forming a series of permanent pools of varying depth for ca. 250 m. Annual rainfall for C. E. Miller Ranch averages between 35–38 cm/year (C. Miller, unpublished data); average temperatures for Valentine (ca. 20 km to the east) range from 32–34 °C in the summer and 15–18 °C in the winter (www.usclimatedata.com).

### Habitat associations

The Sierra Vieja biotic district is located in the Chihuahuan biotic province and is subdivided into two life belts: 1) the Roughland belt which comprises the Sierra Vieja and 2) the Plains belt which comprises the Valentine Plain lowlands that occur between the Sierra Vieja and the Davis Mountains to the east (Dice 1943; York 1949). York (1949) further described and quantified vegetation associations that occur within these life belts in the Sierra Vieja, including seven vegetation associations from the Plains belt (catclaw-cedar, catclaw-tobosa, tobosa-grama, creosote bush-catclaw-blackbrush, mesquite-huisache-blackbrush, yucca-blackbrush-grama, and blackbrush-creosote bush; Fig. 2) and six vegetation associations from the Roughland belt (stream bed, catclaw-grama, grama-bluestem, rock bluff, huisache-lechuguilla, and lechuguilla-beargrass; Fig. 2). The most abundant vegetation associations within the Plains belt at the ranch were the tobosa-grama and catclaw-tobosa associations and the most abundant in the Roughland belt was the lechuguilla-beargrass association (York 1949).

### Data collection

An amphibian and reptile survey was conducted from 3 June–9 July 1948 as part of a large expedition to further understand the biodiversity of the Chihuahuan Desert (Jameson and Flury 1949). Our recent amphibian and reptile surveys were conducted from 2004–2017, with one to six sampling trips per year during the months of May–October. Surveys for amphibians and reptiles were conducted opportunistically at both day and night by hiking through canyons and along trails, searching under rocks, debris, and cover objects, trapping and seining aquatic habitats, and driving roads looking for road-killed, thermoregulating, or actively moving amphibians and reptiles. Recent collections were primarily made by the authors, though additional individuals occasionally participated in surveys as well. Additionally, in May 2007, the Texas Herpetological Society spring field meet was held at C. E. Miller Ranch, in which over 25 amateurs and professionals participated in survey efforts over three days.

Voucher specimens of all species that were encountered were collected and deposited at the Biodiversity Collections (formerly the Texas Natural History Collections) at The University of Texas at Austin or at the Biodiversity Research and Teaching Collections (formerly the Texas Cooperative Wildlife Collections) at Texas A&M University. Specimens from the 1948 survey were all deposited at the Biodiversity Collections. Specimens from recent surveys were collected under a Texas Parks and Wildlife Department Scientific Collecting Permit issued to TJL (#SPR-1097-912); collections were performed under University of Texas IACUC protocol AUP-2015-00106 (and earlier versions of this protocol). Individuals were collected, then euthanized with aqueous chlorotone or benzocaine (amphibians) or via injection of sodium pentobarbital (reptiles). Tissue samples (muscle or liver) were collected from specimens and individuals were then fixed in 10% buffered formalin for a minimum of 48 h, then transferred to 70% ethanol for long-term storage. Species identifications primarily follow those outlined by Crother (2017) and Devitt et al. (2008). Additionally, we recognize the genera *Bufo* (Pauly et al. 2009), *Syrrhophus* (Lynch 1970; T. Devitt, pers. comm.), *Rana* (Yuan et al. 2016), and *Masticophis* (Myers et al. 2017), as well as the species *Salvadora deserticola* (Bogert 1945, 1985).

## Results

**Figure 3.**
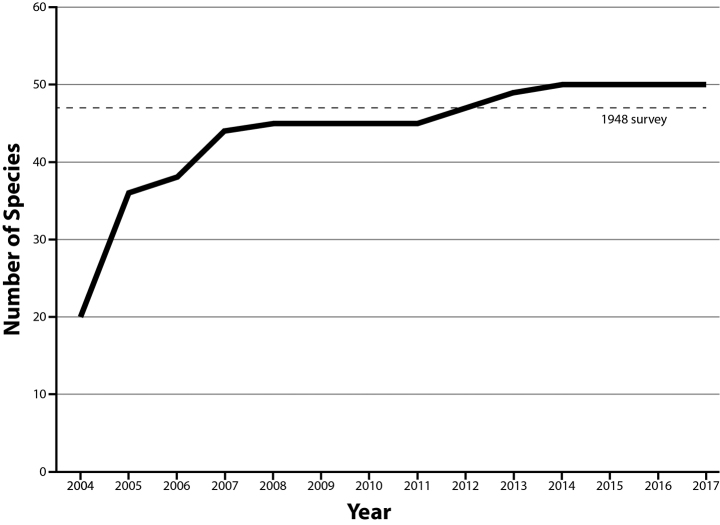
Species accumulation curve for species encountered during recent survey years at C. E. Miller Ranch from 2004–2017. Values indicate total number of species known at the end of each year. The dashed line represents the total number of species detected during the 1948 survey of the site.

**Figure 4.**
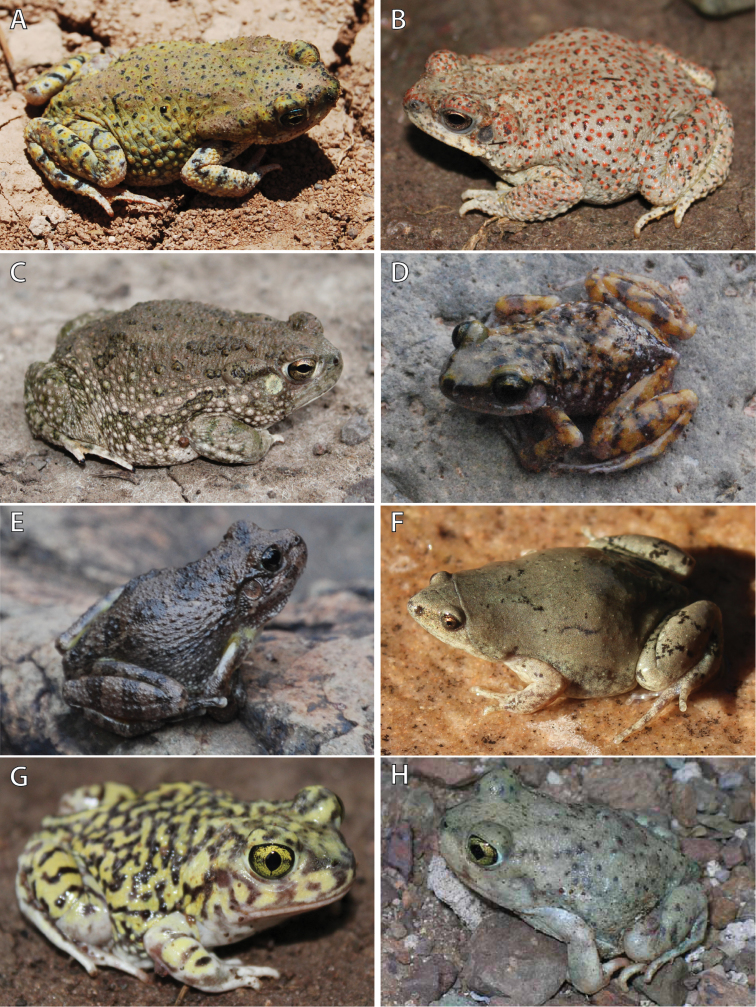
Photos in life of species of anurans collected from C. E. Miller Ranch. **A** Chihuahuan Green Toad (*Bufo debilis*) **B** Red-spotted Toad (*Bufo punctatus*) **C** Texas Toad (*Bufo speciosus*) **D** Cliff Chirping Frog (*Syrrhophus marnockii*) **E** Canyon Treefrog (*Hyla arenicolor*) **F** Western Narrow-mouthed Toad (*Gastrophryne olivacea*) **G** Couch’s Spadefoot (*Scaphiopus couchii*), and **H** Mexican Spadefoot (*Spea multiplicata*). Photos by DRD.

**Figure 5.**
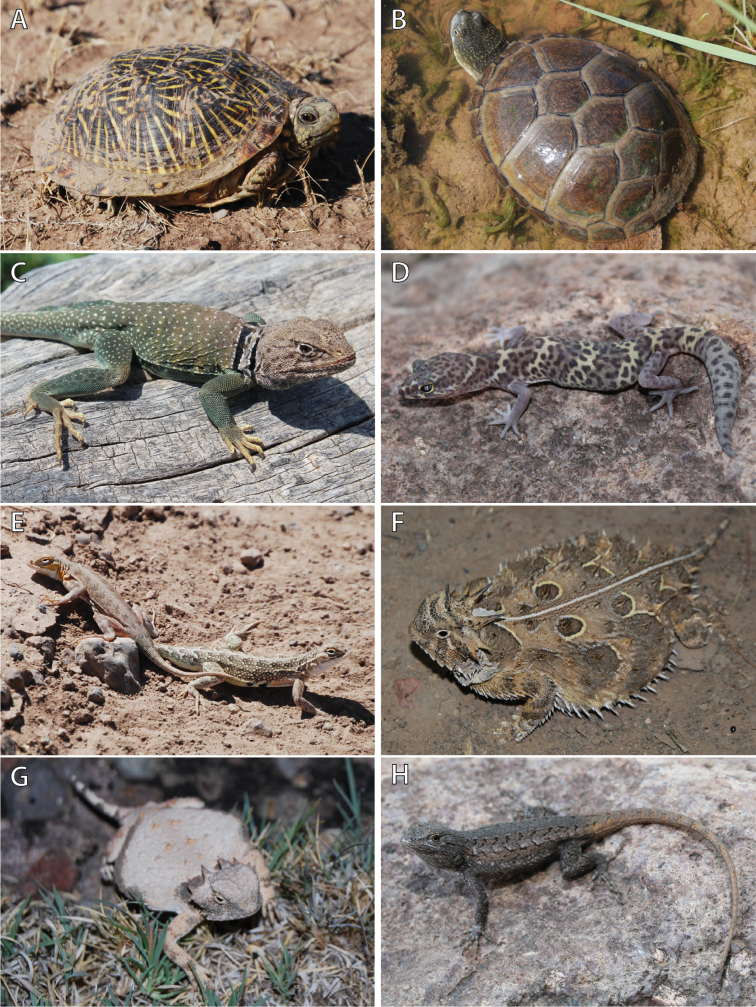
Photos in life of species of turtles and lizards collected from C. E. Miller Ranch. **A** Ornate Box Turtle (*Terrapene ornata*) **B** Yellow Mud Turtle (*Kinosternon flavescens*) **C** Eastern Collard Lizard (*Crotaphytus collaris*) **D** Texas Banded Gecko (*Coleonyx brevis*) **E** Common Lesser Earless Lizard (*Holbrookia maculata*) **F** Texas Horned Lizard (*Phrynosoma cornutum*) **G** Round-tailed Horned Lizard (*Phrynosoma modestum*), and **H** Southwestern Fence Lizard (*Sceloporus cowlesi*). Photos by DRD.

**Figure 6.**
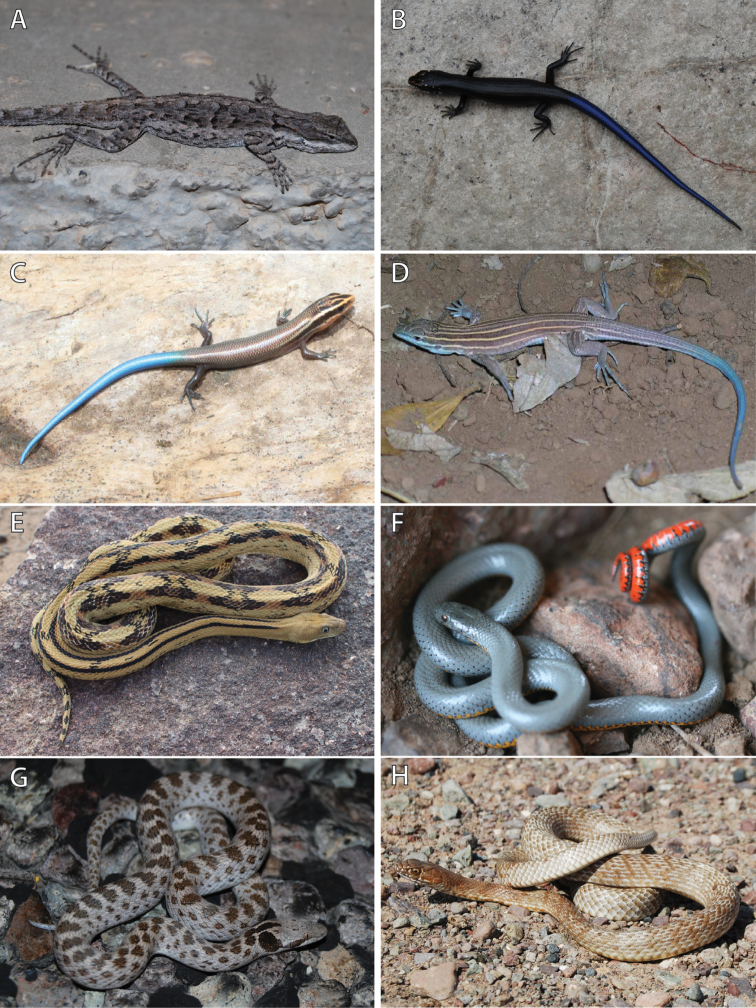
Photos in life of species of lizards and snakes collected from C. E. Miller Ranch. **A** Ornate Tree Lizard (*Urosaurus ornatus*) **B** juvenile Great Plains Skink (*Plestiodon obsoletus*) **C** juvenile Four-lined Skink (*Plestiodon tetragrammus*) **D** Chihuahuan Spotted Whiptail (*Aspidoscelis exsanguis*) **E** Trans-Pecos Ratsnake (*Bogertophis subocularis*) **F** Ring-necked Snake (*Diadophis punctatus*) **G** Chihuahuan Nightsnake (*Hypsiglena jani*), and **H** Coachwhip (*Masticophis flagellum*). Photos by DRD.

**Figure 7.**
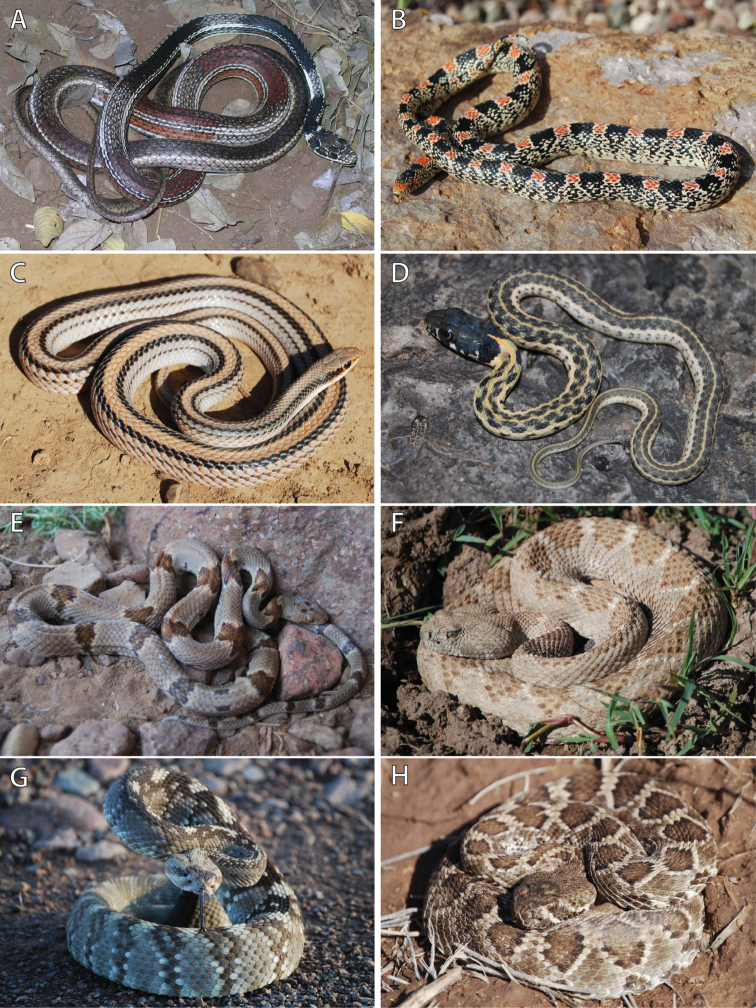
Photos in life of species of snakes collected from C. E. Miller Ranch. **A** Striped Whipsnake (*Masticophis taeniatus*) **B** Long-nosed Snake (*Rhinocheilus lecontei*) **C** Big Bend Patch-nosed Snake (*Salvadora deserticola*) **D** Black-necked Gartersnake (*Thamnophis cyrtopsis*) **E** Chihuahuan Lyresnake (*Trimorphodon vilkinsonii*) **F** Western Diamond-backed Rattlesnake (*Crotalus atrox*) **G** Eastern Black-tailed Rattlesnake (*Crotalus ornatus*), and **H** Mohave Rattlesnake (*Crotalus scutulatus*). Photos by DRD.

A total of 315 specimens comprising 10 species of amphibians and 40 species of reptiles were collected during surveys from 2004–2017 (Fig. 3; Tables 1, 2; Appendix 1). This total was in contrast to the nine species of amphibians and 38 species of reptiles encountered during the survey conducted almost 70 years previous (Jameson and Flury 1949). Of the 50 total species encountered during recent surveys, there were 10 species of anurans, two species of turtles, 14 species of lizards, and 24 species of snakes (Figs 4–7). From 2004–2008, 45 of the 50 species (90%) had been documented on the ranch (Fig. 3). New species were not documented until 2012–2014, when the final five species were collected, despite no noticeable shifts or change in survey effort (Fig. 3). Additionally, six species were collected in 2007 (Fig. 3), four of which correspond to collecting efforts from individuals participating in the Texas Herpetological Society spring field meet. Three species that were collected in 1948 were not encountered during recent surveys: Western Tiger Salamander (*Ambystoma mavortium*), Gray-banded Kingsnake (*Lampropeltis alterna*), and Eastern Patch-nosed Snake (*Salvadora grahamiae*; Tables 1, 2). Six species (two anurans and four snakes) were collected from the study site in recent surveys that were not collected in 1948: Great Plains Toad (*Bufo cognatus*), Texas Toad (*Bufo speciosus*), Chihuahuan Hook-nosed Snake (*Gyalopion canum*), Desert Kingsnake (*Lampropeltis splendida*), Plains Black-headed Snake (*Tantilla nigriceps*), and New Mexico Threadsnake (*Rena dissecta*; Tables 1, 2). In total, 11 species of amphibians (10 frogs, 1 salamander) and 42 species of reptiles (2 turtles, 14 lizards, 26 snakes) have been documented from C. E. Miller Ranch. Species accounts discussing the status and distribution of these species on the ranch are included below.

**Table 1.** Amphibian species from C. E. Miller Ranch and the Sierra Vieja. Species presence during the historic (1948) and recent (2004–2017) surveys are indicated. × = vouchered individuals present, * = species heard, but not collected.

Order	Family	Species Name	Common Name	1948	2004–2017
Anura	Bufonidae	*Bufo cognatus*	Great Plains Toad		×
		*Bufo debilis*	Chihuahuan Green Toad	×	×
		*Bufo punctatus*	Red-spotted Toad	×	×
		*Bufo speciosus*	Texas Toad		×
	Eleutherodactylidae	*Syrrhophus marnockii*	Cliff Chirping Frog	*	×
	Hylidae	*Hyla arenicolor*	Canyon Treefrog	×	×
	Microhylidae	*Gastrophryne olivacea*	Western Narrow-mouthed Toad	×	×
	Ranidae	*Rana berlandieri*	Rio Grande Leopard Frog	×	×
	Scaphiopodidae	*Scaphiopus couchii*	Couch’s Spadefoot	×	×
		*Spea multiplicata*	Mexican Spadefoot	×	×
Caudata	Ambystomatidae	*Ambystoma mavortium*	Western Tiger Salamander	×	

**Table 2.** Reptile species from C. E. Miller Ranch and the Sierra Vieja. Species presence during the historic (1948) and recent (2004–2017) surveys are indicated. × = vouchered individuals present, # = vouchered individual collected in 1947, but not seen in 1948.

Order	Family	Species Name	Common Name	1948	2004–2017
Testudines	Emydidae	*Terrapene ornata*	Ornate Box Turtle	×	×
	Kinosternidae	*Kinosternon flavescens*	Yellow Mud Turtle	×	×
Squamata	Crotaphytidae	*Crotaphytus collaris*	Eastern Collared Lizard	×	×
	Gekkonidae	*Coleonyx brevis*	Texas Banded Gecko	×	×
	Phrynosomatidae	*Cophosaurus texanus*	Greater Earless Lizard	×	×
		*Holbrookia maculata*	Common Lesser Earless Lizard	×	×
		*Phrynosoma cornutum*	Texas Horned Lizard	×	×
		*Phrynosoma modestum*	Round-tailed Horned Lizard	×	×
		*Sceloporus cowlesi*	Southwestern Fence Lizard	×	×
		*Sceloporus poinsettii*	Crevice Spiny Lizard	×	×
		*Urosaurus ornatus*	Ornate Tree Lizard	×	×
	Scincidae	*Plestiodon obsoletus*	Great Plains Skink	×	×
		*Plestiodon tetragrammus*	Four-lined Skink	×	×
	Teiidae	*Aspidoscelis exsanguis*	Chihuahuan Spotted Whiptail	×	×
		*Aspidoscelis inornata*	Little Striped Whiptail	×	×
		*Aspidoscelis tesselata*	Common Checkered Whiptail	×	×
	Colubridae	*Bogertophis subocularis*	Trans-Pecos Ratsnake	×	×
		*Diadophis punctatus*	Ring-necked Snake	×	×
		*Gyalopion canum*	Chihuahuan Hook-nosed Snake		×
		*Heterodon kennerlyi*	Mexican Hog-nosed Snake	×	×
		*Hypsiglena jani*	Chihuahuan Nightsnake	×	×
		*Lampropeltis alterna*	Gray-banded Kingsnake	×	
		*Lampropeltis splendida*	Desert Kingsnake		×
		*Masticophis flagellum*	Coachwhip	×	×
		*Masticophis taeniatus*	Striped Whipsnake	×	×
		*Pituophis catenifer*	Gophersnake	×	×
		*Rhinocheilus lecontei*	Long-nosed Snake	×	×
		*Salvadora deserticola*	Big Bend Patch-nosed Snake	×	×
		*Salvadora grahamiae*	Eastern Patch-nosed Snake	×	
		*Sonora semiannulata*	Western Groundsnake	×	×
		*Tantilla hobartsmithi*	Smith’s Black-headed Snake	×	×
		*Tantilla nigriceps*	Plains Black-headed Snake		×
		*Thamnophis cyrtopsis*	Black-necked Gartersnake	×	×
		*Thamnophis marcianus*	Checkered Gartersnake	×	×
		*Trimorphodon vilkinsonii*	Chihuahuan Lyresnake	×	×
	Leptotyphlopidae	*Rena dissecta*	New Mexico Threadsnake		×
		*Rena humilis*	Western Threadsnake	×	×
	Viperidae	*Crotalus atrox*	Western Diamond-backed Rattlesnake	×	×
		*Crotalus lepidus*	Rock Rattlesnake	×	×
		*Crotalus ornatus*	Eastern Black-tailed Rattlesnake	×	×
		*Crotalus scutulatus*	Mohave Rattlesnake	#	×
		*Crotalus viridis*	Prairie Rattlesnake	#	×

### Species accounts

#### Class Amphibia, Order Anura

##### Family Bufonidae

***Bufo cognatus*** Say, 1823

Great Plains Toad

Five individuals were collected during recent surveys, primarily from the tobosa-grama and catclaw-tobosa associations on the Valentine Plain. Previously, *B. cognatus* was not known from the ranch. The failure to detect this species on the ranch in 1948 despite seemingly favorable environmental conditions (i.e., heavy rains the preceding week) may suggest that this species has increased its range or abundance in the area. *Bufo cognatus* appears to be less common in the area than *B. speciosus*, though both species occupy similar habitats in the tobosa-grama association.

***Bufo debilis*** Girard, 1854

Chihuahuan Green Toad (Fig. 4A)

Fourteen individuals were collected during recent surveys, most from the tobosa-grama and catclaw-tobosa associations on the Valentine Plain. This species appears to be common in the area and is frequently observed and heard calling in small ephemeral pools in the tobosa-grama association or along Wild Horse Draw after rains. *Bufo debilis* was frequently encountered and appeared similarly abundant during the 1948 survey (Jameson and Flury 1949).

***Bufo punctatus*** Baird & Girard, 1852

Red-spotted Toad (Fig. 4B)

Fifteen individuals were collected during recent surveys, all from rocky canyons in the Sierra Vieja and from where these canyons empty out onto the Valentine Plain. This species appears to be common in these rocky habitats and was primarily found in the stream bed and catclaw-cedar associations, similar to that in the 1948 survey (Jameson and Flury 1949). *Bufo punctatus* was previously reported from dugout cattle tanks in the tobosa-grama association (Jameson and Flury 1949); however, we only detected individuals in the Valentine Plain near the mouths of Sierra Vieja canyons.

***Bufo speciosus*** Girard, 1854

Texas Toad (Fig. 4C)

Ten specimens were collected from the Valentine Plain during recent surveys and represent one of the two amphibians previously unknown from the ranch. The failure to detect this species on the ranch in 1948 despite seemingly favorable environmental conditions (i.e., heavy rains the preceding week; Jameson and Flury 1949) may suggest that this species has increased its range or abundance in the area. *Bufo speciosus* appears to be more common than *B. cognatus* in the area, though both species occupy similar habitats in the tobosa-grama association.

##### Family Eleutherodactylidae

***Syrrhophus marnockii*** Cope, 1878

Cliff Chirping Frog (Fig. 4D)

A single individual was collected in the Sierra Vieja during recent surveys (Owen et al. 2014). This species was frequently heard calling during the 1948 survey on rainy nights, but no individuals were collected. During recent surveys, this species was frequently heard calling in canyons in the Sierra Vieja, and individuals were observed only twice, once in the stream bed association and once in the rock bluff association.

##### Family Hylidae

***Hyla arenicolor*** Cope, 1866

Canyon Treefrog (Fig. 4E)

Seven individuals were collected during recent surveys, all from the Sierra Vieja, except one individual that was collected at Glidewell Pond in the Valentine Plain. These results are similar to those reported in Jameson and Flury (1949), with this species being fairly common along springs and pools in the stream bed association that line the canyons in the Sierra Vieja. The individual from Glidewell Pond likely moved down from the Sierra Vieja and through the catclaw-cedar association that occurs along the mouths of canyons and through the catclaw-tobosa association to Glidewell Pond. This species is also periodically found around the irrigated landscape of the ranch headquarters, ca. 1.5 km east of the Sierra Vieja.

##### Family Microhylidae

***Gastrophryne olivacea*** Hallowell, 1857

Western Narrow-mouthed Toad (Fig. 4F)

Seven specimens were collected during recent surveys. Most of these specimens came from dugout ponds in the Valentine Plain near the eastern slopes of the Sierra Vieja, but one individual was collected in the stream bed association in Box Canyon, which suggests that individuals may move into the canyons from the Valentine Plain on occasion. Unlike our detection of *G. olivacea* primarily from the Valentine Plains, Jameson and Flury (1949) indicated *G. olivacea* were collected in roughly equal numbers from both the Valentine Plain and Sierra Vieja.

##### Family Ranidae

***Rana berlandieri*** Baird, 1859

Rio Grande Leopard Frog

Six specimens were collected during recent surveys: five from the Sierra Vieja and one from the Valentine Plain. Within the Sierra Vieja, this species can be found in the stream bed association along springs, spring runs, and pools. Jameson and Flury (1949) reported *R. berlandieri* in equal abundance in both the Sierra Vieja and the Valentine Plain; however, despite extensive surveys around suitable habitat in the Valentine Plain (e.g., dugout ponds that occur along the eastern edge of the Sierra Vieja), only a single individual has been observed in a dugout pond along Wild Horse Draw.

##### Family Scaphiopodidae

***Scaphiopus couchii*** Baird, 1854

Couch’s Spadefoot (Fig. 4G)

Nine specimens were collected during recent surveys, with seven collected in the Valentine Plain and two collected from the Sierra Vieja. Despite individuals being collected in both the Plains and Roughland life belts, this species is infrequently encountered in the Sierra Vieja. Jameson and Flury (1949) reported individuals only from the Valentine Plain. Individuals occasionally may disperse into Sierra Vieja canyons, though breeding activity appears to exclusively take place in shallow, ephemeral pools that fill after heavy rains in the tobosa-grama and catclaw-tobosa associations of the Valentine Plain.

***Spea multiplicata*** (Cope, 1863)

Mexican Spadefoot (Fig. 4H)

Sixteen specimens were collected during recent surveys, all from the Valentine Plain. Jameson and Flury (1949) reported individuals collected from the Valentine Plain, but also collected individuals from the stream bed and catclaw-tobosa associations of the Sierra Vieja. Those individuals collected in the Sierra Vieja likely represent individuals dispersing into the canyons from the Valentine Plain. This species appears common, especially after mid- to late-summer rains and is frequently found in shallow, ephemeral pools in the tobosa-grama vegetation association in the Valentine Plain.

#### Class Amphibia, Order Caudata

##### Family Ambystomatidae

***Ambystoma mavortium*** Baird, 1850

Western Tiger Salamander

This species was not detected during recent surveys despite extensive survey effort in potential habitats (e.g., dugout ponds in the Valentine Plain) and is presumed locally extirpated. In 1948, a large lot (172 individuals) of larval specimens was collected from a single dugout pond in the tobosa-grama association on the eastern side of the Sierra Vieja.

#### Class Reptilia, Order Testudines

##### Family Emydidae

***Terrapene ornata*** (Agassiz, 1857)

Ornate Box Turtle (Fig. 5A)

Three specimens were collected during recent surveys, all from the Valentine Plain. This species likely occurs throughout the various associations in the Valentine Plain as suggested in Jameson and Flury (1949), but has been most frequently observed in the tobosa-grama and catclaw-tobosa associations during recent surveys.

##### Family Kinosternidae

***Kinosternon flavescens*** (Agassiz, 1857)

Yellow Mud Turtle (Fig. 5B)

Twenty-five specimens were collected in recent surveys as part of a separate project studying the natural history of this species in the region. All specimens were taken from either natural or artificial ponds in the Valentine Plain. These turtles seem to be abundant at these sites and are occasionally found in temporary, ephemeral pools after heavy rains. Many of these sites occur in the tobosa-grama, catclaw-tobosa, and catclaw-cedar associations, similar to localities reported in Jameson and Flury (1949).

#### Class Reptilia, Order Squamata

##### Family Crotaphytidae

***Crotaphytus collaris*** (Say, 1823)

Eastern Collared Lizard (Fig. 5C)

Three specimens were collected in recent surveys: two from the Sierra Vieja and one from the Valentine Plain. Within the Sierra Vieja, *C. collaris* has been observed in the stream bed and catclaw-grama associations and in the Valentine Plain it has been observed in the rocky areas near the eastern slopes of the Sierra Vieja. Jameson and Flury (1949) reported *C. collaris* from rocky areas in similar habitat associations in roughly equal proportions in both the Sierra Vieja and Valentine Plain.

##### Family Gekkonidae

***Coleonyx brevis*** Stejneger, 1893

Texas Banded Gecko (Fig. 5D)

Seven specimens were collected in recent surveys: six from the Sierra Vieja and one from the Valentine Plain near the mouth of ZH Canyon. All of these specimens from the Sierra Vieja were collected in the stream bed association, and the single individual from the Valentine Plain was taken in the catclaw-cedar association where large rocks and boulders are present. Jameson and Flury (1949) reported this species in rocky areas from both the Sierra Vieja and the Valentine Plain, in roughly equal proportions.

##### Family Phrynosomatidae

***Cophosaurus texanus*** Troschel, 1852

Greater Earless Lizard

Fifteen specimens were collected during recent surveys, all from areas along the eastern slopes of the Sierra Vieja and nearby areas in the Valentine Plain. This species appears common in rocky habitats where it is frequently encountered perching on top of large rocks. Within the Sierra Vieja, individuals have been encountered in the stream bed and catclaw-grama associations, and in the Valentine Plain, individuals were primarily encountered in the creosote bush-catclaw-blackbrush association. Jameson and Flury (1949) reported *C. texanus* from similar habitat associations, however, the majority of their specimens collected from the Valentine Plain were taken in the catclaw-cedar association.

***Holbrookia maculata*** Girard, 1851

Common Lesser Earless Lizard (Fig. 5E)

Six specimens were collected during recent surveys, all from the tobosa-grama association on the Valentine Plain. Individuals appeared abundant in these sandy habitats. Jameson and Flury (1949) reported individuals from additional habitat associations, though the majority of specimens they collected were taken from the catclaw-tobosa association.

***Phrynosoma cornutum*** (Harlan, 1825)

Texas Horned Lizard (Fig. 5F)

Six specimens were collected during recent surveys, all from the Valentine Plain. *Phrynosoma cornutum* appears widespread throughout most habitats in the Valentine Plain, especially in the tobosa-grama and catclaw-tobosa associations, similar to reports in Jameson and Flury (1949). Despite declines in the abundance of this species throughout its range (Price 1990), it still remains common throughout this study site.

***Phrynosoma modestum*** Girard, 1852

Round-tailed Horned Lizard (Fig. 5G)

Four specimens were collected during recent surveys from both the Sierra Vieja and Valentine Plain. Individuals appear to be abundant in the rocky habitats along the eastern slopes of the Sierra Vieja where they have been detected in the stream bed and catclaw-grama associations Additionally, this species was found in the rockier portions of the catclaw-tobosa and creosote bush-catclaw-blackbrush associations in the Valentine Plain. Jameson and Flury (1949) reported *P. modestum* from additional habitats in the Valentine Plain, though they only cite a single specimen from the Sierra Vieja.

***Sceloporus cowlesi*** Lowe & Norris, 1956

Southwestern Fence Lizard (Fig. 5H)

Seven specimens were collected during recent surveys, all from the Valentine Plain. Individuals were primarily encountered in the catclaw-tobosa and tobosa-grama associations where they were commonly observed on vertical structure (e.g., fence posts, yuccas, catclaw), similar to habitat associations reported in Jameson and Flury (1949). Although Jameson and Flury (1949) reported a single individual from the Sierra Vieja, we observed no individuals there during our recent surveys.

***Sceloporus poinsettii*** Baird & Girard, 1852

Crevice Spiny Lizard

Only a single photo voucher of this species exists from recent surveys from an abandoned stone structure in Fort Holland in the Sierra Vieja. During historic surveys this species was frequently encountered in rock crevices throughout stream bed, catclaw-grama, and rock bluff associations in the Sierra Vieja (Jameson and Flury 1949). Despite extensive survey efforts in the canyons of the Sierra Vieja, no other individuals have been collected.

***Urosaurus ornatus*** (Baird & Girard, 1852)

Ornate Tree Lizard (Fig. 6A)

Nine specimens were collected during recent surveys, all from the catclaw-grama association in the Sierra Vieja. This species was frequently encountered at Fort Holland where individuals would be observed on the walls and rafters of buildings. Jameson and Flury (1949) reported *U. ornatus* from additional habitat associations in the Sierra Vieja, but also in rocky areas in the catclaw-cedar association along the eastern edge of the Sierra Vieja in the Valentine Plain.

##### Family Scincidae

***Plestiodon obsoletus*** Baird & Girard, 1852

Great Plains Skink (Fig. 6B)

Six specimens were collected in recent surveys from both the Sierra Vieja and Valentine Plain. Individuals primarily occur in the catclaw-grama and stream bed associations in the Sierra Vieja and the catclaw-cedar and catclaw-tobosa associations in the Valentine Plain where large rocky areas exist, similar to locations reported in Jameson and Flury (1949).

***Plestiodon tetragrammus*** Baird, 1859

Four-lined Skink (Fig. 6C)

Four specimens were collected in recent surveys, all from the Sierra Vieja. This species is infrequently encountered and all occurrences of individuals have been reported from the stream bed and catclaw-grama associations, similar to locations listed in Jameson and Flury (1949).

##### Family Teiidae

***Aspidoscelis exsanguis*** (Lowe, 1956)

Chihuahuan Spotted Whiptail (Fig. 6D)

Sixteen specimens were collected in recent surveys, from both the Valentine Plain and the Sierra Vieja, where this species appears to be more abundant. Jameson and Flury (1949) reported that this species was primarily in the Sierra Vieja and in rocky regions of the Valentine Plain along the eastern edge of the Sierra Vieja; however, we have collected this species in areas dominated by sandy soils ca. 6 km east of the Sierra Vieja in the Valentine Plain. Within the Sierra Vieja, this species is frequently observed in the catclaw-grama association.

***Aspidoscelis inornata*** (Baird, 1859)

Little Striped Whiptail

Fifteen specimens were collected in recent surveys, from both the Sierra Vieja and Valentine Plain. Jameson and Flury (1949) reported that this species appeared to be restricted to the Valentine Plain. While individuals do appear to be more abundant in the catclaw-tobosa association in the Valentine Plain, numerous individuals were taken in catclaw-grama association surrounding Fort Holland.

***Aspidoscelis tesselata*** (Say, 1823)

Common Checkered Whiptail

Eight specimens were collected in recent surveys, from both the Sierra Vieja and Valentine Plain. This species has been encountered in many different habitats, including tobosa-grama, catclaw-cedar, stream bed, and catclaw-grama associations. Jameson and Flury (1949) also collected *A. tesselata* from the Valentine Plain and Sierra Vieja, though encountered individuals in additional habitat associations within both life belts.

##### Family Colubridae

***Bogertophis subocularis*** (Brown, 1901)

Trans-Pecos Ratsnake (Fig. 6E)

Two individuals were collected during recent surveys, one from the Sierra Vieja and one from the Valentine Plain. Within the Sierra Vieja, one individual was collected in the stream bed association at the mouth of ZH Canyon. Within the Valentine Plain, one individual was collected from the vicinity of the ranch headquarters in the catclaw-tobosa association. Individuals have been found throughout the Sierra Vieja (stream bed, catclaw-grama, and rock bluff associations) and Valentine Plain (catclaw-tobosa and catclaw-cedar) adjacent to the Sierra Vieja. Six individuals were observed during a one-week span in June 2007, including three found while walking through ZH and Box canyons between 2100–0000 h. Two of those individuals were found climbing in vegetation (*Quercus* sp. and *Acacia greggi*; Pauly and LaDuc 2008); an additional animal was found 1 m above the ground in a catclaw on a subsequent survey. Jameson and Flury (1949) reported *B. subocularis* from similar habitats in both the Sierra Vieja and Valentine Plain.

***Diadophis punctatus*** (Linnaeus, 1766)

Ring-necked Snake (Fig. 6F)

Two individuals were collected from the ranch during recent surveys, with our first specimen being found nine years into our survey. One individual was found in the stream bed association in Box Canyon in the Sierra Vieja and the second individual was found at the ranch headquarters in the catclaw-tobosa association of the Valentine Plain. An additional specimen was collected crossing a dirt road in the yucca-tobosa association, just east of the ranch, which may suggest that *D. punctatus* has a broader range in the Valentine Plain. None of the specimens encountered possessed a nuchal ring. Jameson and Flury (1949) reported this species solely from the stream bed association in the Sierra Vieja.

***Gyalopion canum*** Cope, 1860

Chihuahuan Hook-nosed Snake

A single specimen was collected during recent surveys and represents a new species for the ranch that was not detected in 1948. The female snake was collected just after midnight in June 2012 in the catclaw-grama association between the rocky bluffs and stream bed of Fox Hollow in the Sierra Vieja. Because this species is represented by this single individual, we consider this species among the most cryptic species of snake at the ranch.

***Heterodon kennerlyi*** Kennicott, 1860

Mexican Hog-nosed Snake

Three individuals were collected during recent surveys from both the Valentine Plain and the Sierra Vieja. Several individuals have been observed in the catclaw-tobosa and tobosa-grama associations in the Valentine Plain. One individual was collected in a peculiarly sandy portion of the catclaw-grama association in the Sierra Vieja (adjacent to Fort Holland), suggesting that this species may be tied to soils rather than vegetation associations on the ranch. Jameson and Flury (1949) reported *H. kennerlyi* only from the Valentine Plain, and hypothesized this species was restricted to the associations therein.

***Hypsiglena jani*** (Dugès, 1865)

Chihuahuan Nightsnake (Fig. 6G)

Four specimens were collected during recent surveys from both the Valentine Plain and the Sierra Vieja. This species is likely distributed across the entire ranch and most of the vegetation associations. Individuals have been collected under debris in the catclaw-tobosa association, crawling in the open in the stream bed association, or across roads at night in the yucca-tobosa association. One individual collected at night just east of the property had recently ingested two *Aspidoscelis inornata*, representing a new maximum prey/predator mass ratio (Davis and LaDuc 2017). Jameson and Flury (1949) report that *H. jani* appeared to be restricted to rocky areas, though our observations of individuals have occurred in habitats devoid of rocks.

***Lampropeltis alterna*** (Brown, 1901)

Gray-banded Kingsnake

This species has not been encountered during the recent surveys, despite exhaustive surveys over a decade at the same locality where the single female snake was collected in July 1948: the stream bed and catclaw-grama associations at the mouth of Fox Hollow in the Sierra Vieja. Further, this habitat is similar to the area around Fort Holland and ZH Canyon that has been intensively sampled, producing no specimens for the past 14 years.

***Lampropeltis splendida*** (Baird & Girard, 1853)

Desert Kingsnake

This species was not encountered on the ranch in 1948, but multiple individuals have been seen in recent surveys in the tobosa-grama and catclaw-tobosa associations in the Valentine Plain, where *L. splendida* is likely restricted. A gravid female was collected in June 2005 under debris (tobosa-grama association) and laid five eggs, which all subsequently hatched. Two hatchlings were preserved as vouchers; the remaining hatchlings and the female were later released. Another individual was collected at 2 Section Tank in the tobosa-grama association and two additional individuals were observed closer to the Sierra Vieja in the rocky catclaw-tobosa association of the Valentine Plain.

***Masticophis flagellum*** (Shaw, 1802)

Coachwhip (Fig. 6H)

Five specimens were collected during recent surveys from the Sierra Vieja and Valentine Plain. Jameson and Flury (1949) commented that *M. flagellum* being more abundant in the Valentine Plain than the Sierra Vieja, though we observed this species in roughly equal proportions between these two regions. The three animals from the Sierra Vieja were collected from the catclaw-grama association surrounding Fort Holland, the stream bed of ZH Canyon, and the lechuguilla-beargrass association on the mesa above Fort Holland. From the Valentine Plain, one animal was collected at the mouth of ZH Canyon in the cedar-catclaw association and another from the catclaw-tobosa alluvial plain east of the ranch house. This species is likely widely distributed across the ranch and in most habitat associations.

***Masticophis taeniatus*** (Hallowell, 1852)

Striped Whipsnake (Fig. 7A)

A single individual was collected in a funnel trap at Fort Holland in the catclaw-grama association of the Sierra Vieja. One individual was captured while it was drinking from the stone tank at the mouth of ZH Canyon (catclaw-cedar association), but not collected. Other individuals have been seen but not collected due to the speed of the snakes and the dense nature of both the vegetation and the collectors. This species is likely found across all of the rocky associations of the Sierra Vieja, as well as those associations in the Valentine Plain along the eastern border of the Sierra Vieja. Similarly, Jameson and Flury (1949) reported that *M. taeniatus* was collected only from the Sierra Vieja in rocky habitat associations.

***Pituophis catenifer*** (Blainville, 1835)

Gophersnake

Despite only two specimens having been collected during recent surveys, this species has been seen (and not collected) from multiple areas across the property and is likely widely distributed among most habitat associations. The two specimens collected were each found adjacent to a human dwelling within the catclaw-tobosa association of the Valentine Plain; several additional snakes have been found near these dwellings, likely attracted because of the availability of small prey associated with farms and ranches (i.e., rodents, chickens). Individuals have also been caught, marked, and released from the catclaw-grama association in Box Canyon in the Sierra Vieja, crossing several roads through the catclaw-tobosa alluvial fans, and near human structures in the tobosa-grama association in the Valentine Plain. Jameson and Flury (1949) similarly remarked on the broad distribution of *P. catenifer* across the ranch in both the Valentine Plain and Sierra Vieja.

***Rhinocheilus lecontei*** Baird & Girard, 1853

Long-nosed Snake (Fig. 7B)

Only a single specimen was collected during recent surveys in the Valentine Plain. This individual was found at night while it was crossing the road within the catclaw-tobosa association. Despite extensive surveys, *R. lecontei* has not been encountered in the Sierra Vieja in recent years. Jameson and Flury (1949) reported on four individuals collected from the ranch: three from the catclaw-grama association in the Sierra Vieja and one from the catclaw-tobosa association of the Valentine Plain.

***Salvadora deserticola*** Schmidt, 1940

Big Bend Patch-nosed Snake (Fig. 7C)

This species has been collected during recent surveys in both the Valentine Plain and the Sierra Vieja. Two specimens were collected in and directly adjacent to the Sierra Vieja: one was collected in the catclaw-cedar association at the mouth of ZH Canyon and the other within catclaw-grama association at Fort Holland. Two additional specimens were collected from the Valentine Plain: one in the catclaw-tobosa association just east of the ranch house and the other in the tobosa-grama association east of Wild Horse Draw. This widespread distribution across the property was similar to that reported in Jameson and Flury (1949).

***Salvadora grahamiae*** Baird & Girard, 1853

Eastern Patch-nosed Snake

No specimens of this species have been seen or collected during the recent surveys, though several were collected in 1948 (Jameson and Flury 1949). This species was restricted to rocky habitats in the Sierra Vieja and Valentine Plain during the 1948 surveys, but despite many hours of work in these rocky areas, particularly ZH and Box canyons, the only *Salvadora* that has been collected has been *S. deserticola*.

***Sonora semiannulata*** Baird & Girard, 1853

Western Groundsnake

Four specimens were collected in recent surveys: three from the catclaw-grama association around Fort Holland in the Sierra Vieja and a single specimen from the tobosa-grama association in the Valentine Plain. This species is probably more widely distributed than our collections might indicate. Jameson and Flury (1949) reported a single individual from the catclaw-grama association in the Sierra Vieja.

***Tantilla hobartsmithi*** Taylor, 1936

Smith’s Black-headed Snake

This species has been found in both the Valentine Plain and the Sierra Vieja. Within the Valentine Plain, two specimens were collected under debris within the catclaw-tobosa association and a third specimen was collected under a board at 96 Tank within the creosote bush-catclaw-blackbrush association along the eastern slopes of the Sierra Vieja. In the Sierra Vieja, one individual was collected at Fort Holland in the catclaw-grama association. Jameson and Flury (1949) reported collecting a single individual near the ranch house in the tobosa-grama association of the Valentine Plain.

***Tantilla nigriceps*** Kennicott, 1860

Plains Black-headed Snake

Two specimens were collected during recent surveys, both in the sandy tobosa-grama association in the Valentine Plain. One individual was collected under debris near a storage building while the second was found at night moving on the ground. Jameson and Flury (1949) did not detect this species during previous surveys.

***Thamnophis cyrtopsis*** (Kennicott, 1860)

Black-necked Gartersnake (Fig. 7D)

Eight individuals were collected during recent surveys, all from the Sierra Vieja, except one that was collected away from the main canyons at 96 Tank in the creosote bush-catclaw-blackbrush association of the Valentine Plain. This species is most commonly encountered in close proximity to the springs and pools in the stream bed association in the Sierra Vieja but has also been collected in the catclaw-grama association surrounding Fort Holland. Jameson and Flury (1949) reported *T. cyrtopsis* from similar locations.

***Thamnophis marcianus*** (Baird & Girard, 1853)

Checkered Gartersnake

Six individuals were collected during recent surveys, all from the tobosa-grama, catclaw-tobosa, and creosote bush-catclaw-blackbrush associations in the Valentine Plain. Within these habitats, it is usually found in close proximity to water, whether permanent or ephemeral tanks. One dead and rotting individual was found in a large pool in ZH Canyon in the Sierra Vieja (not vouchered), which may suggest that *T. marcianus* is more widespread across the property than *T. cyrtopsis*. *Thamnophis marcianus* was reported to be found in similar habitats in Jameson and Flury (1949).

***Trimorphodon vilkinsonii*** Cope, 1886

Chihuahuan Lyresnake (Fig. 7E)

Three individuals were collected from the stream bed association of the Sierra Vieja (Box Canyon, Cottonwood Canyon, and ZH Canyon), and one additional animal was captured, bled, and released following injection of a microchip. Two of these animals were found on the ground in the stream bed association and the other two snakes were found climbing in vegetation, 1–3 m above ground (Davis et al. 2008). An additional animal was photographed (not vouchered) by the ranch owners at the ranch house in 2015, ca. 1 km east of the Sierra Vieja in the catclaw-grama association, suggesting that *T. vilkinsonii* occurs in rocky areas of the Valentine Plain. A single individual was collected during previous surveys, a snake found in the stream bed association in the Sierra Vieja (Jameson and Flury 1949).

##### Family Leptotyphlopidae

***Rena dissecta*** (Cope, 1896)

New Mexico Threadsnake

Two individuals were collected from the stream bed association in the Sierra Vieja (Cottonwood Canyon and ZH Canyon) during recent surveys, and this species was not found in the 1948 survey. Because two individuals of *R. humilis* were found in the stream bed association in 1948 (Jameson and Flury 1949), the two recently collected specimens of *R. dissecta* now provide evidence that both species of *Rena* live in close sympatry in the Sierra Vieja.

***Rena humilis*** (Baird & Girard, 1853)

Western Threadsnake

A single individual was collected from the ranch during recent surveys in the catclaw-grama association at Fort Holland in the Sierra Vieja. *Rena humilis* are rarely encountered, though we agree with Jameson and Flury (1949) in that this species may be found throughout the Sierra Vieja as well as rocky associations in the Valentine Plain.

##### Family Viperidae

***Crotalus atrox*** Baird & Girard, 1853

Western Diamond-backed Rattlesnake (Fig. 7F)

Nine individuals of this common species were collected during recent surveys. In descending order of encounter frequency, individuals of *C. atrox* were collected and observed throughout the catclaw-tobosa, tobosa-grama, catclaw-cedar, and creosote bush-catclaw-blackbrush vegetation associations on the Valentine Plain; a single specimen was collected in the Sierra Vieja at Fort Holland in the catclaw-grama vegetation association. Jameson and Flury (1949) commented on the scarcity of *C. atrox* on the ranch during the 1948 survey, finding individuals only in the creosote bush-catclaw-blackbrush association of the Valentine Plain.

***Crotalus lepidus*** (Kennicott, 1861)

Rock Rattlesnake

Only two individuals have been seen since 2005: one adult male was collected in May 2007 from the catclaw-grama association of the Sierra Vieja, and another animal escaped a collector in a south facing talus slope in ZH Canyon in June 2005. This is most infrequently observed species of rattlesnake on the ranch, despite being a commonly encountered animal in the Davis Mountains (ca. 64 air km to the east) and the Indio (Eagle) Mountains (ca. 32 air km to the northwest; TJL, unpubl. data). Jameson and Flury (1949) reported *C. lepidus* from both the catclaw-grama and stream bed associations of the Sierra Vieja.

***Crotalus ornatus*** Hallowell, 1854

Ornate Black-tailed Rattlesnake (Fig. 7G)

This is the mostly commonly encountered rattlesnake in the Sierra Vieja. Only two individuals have been vouchered, but four additional animals were captured, marked, and released. This species was found in the stream bed and catclaw-grama associations surrounding Fort Holland and both Box and ZH canyons in the Sierra Vieja. *Crotalus ornatus* was also found in the catclaw-tobosa association and the alluvial fans spreading east from the mountains on the Valentine Plain. Habitat associations where we have found *C. ornatus* are similar to those reported in Jameson and Flury (1949).

***Crotalus scutulatus*** (Kennicott, 1861)

Mohave Rattlesnake (Fig. 7H)

This species is commonly encountered in the sandier catclaw-tobosa and tobosa-grama associations on the Valentine Plain and we have collected sixteen specimens over the course of our recent surveys. W. F. Blair collected a single specimen from an earlier trip to the ranch in July 1947 from the tobosa-gram association of the Valentine Plain (Jameson and Flury 1949), but no individuals were collected as part of the 1948 survey. The consistent number of individuals encountered across our recent survey efforts would suggest population numbers are greater now than in the 1940s, despite any noticeable shifts in vegetative distribution or composition over the last 70 years. An instance of climbing in this species was noted at the ranch by Davis and Cardwell (2017).

***Crotalus viridis*** (Rafinesque, 1818)

Prairie Rattlesnake

This species is infrequently encountered on the ranch, with only three observed on the ranch property (one vouchered) in 2007. Two additional specimens were collected from the main dirt road leading into the ranch from Valentine (once in 2009, another in 2014). All observations of *C. viridis* are restricted to the sandy soils of the tobosa-grama or yucca-grama associations. C. E. Miller, Jr. collected a single specimen from an earlier trip to the ranch in June 1947 from the tobosa-grama association in the Valentine Plain (Jameson and Flury 1949), but no individuals were collected as part of the 1948 survey.

## Discussion

The species composition of amphibians and reptiles encountered during recent years has remained remarkably similar to that observed during the historic 1948 survey. Out of the 47 species of amphibians and reptiles detected in 1948, 44 species (93.6%) have been collected during recent surveys, and many of the localities where specimens were collected in 1948 remain similar to localities where species have been collected in recent years. These results highlight the critical importance of land stewardship in maintaining species diversity at this study site. Additionally, six of the 50 species (12%) encountered in recent years were not collected during the 1948 survey. One of these species, *Lampropeltis splendida*, was collected in areas near, but just off, the study site in 1948, and two anuran species, *Bufo cognatus* and *B. speciosus*, were collected in 1948 along the Rio Grande near Porvenir, ca. 20 km (air) west of the Sierra Vieja. The remaining three species (*Gyalopion canum*, *Tantilla nigriceps*, and *Rena dissecta*) were never encountered in 1948. All of these six species that were undetected in 1948 occur in this region and should have been expected to occur at the study site, but may not have been encountered due to unfavorable environmental conditions from June–July 1948, their cryptic nature, or existed in low abundances making detection difficult. Jameson and Flury (1949) reported that heavy rains fell on the ranch the week prior to their surveys. Both *B. cognatus* and *B. speciosus* occupy grassland habitats and use ephemeral pools as breeding habitats similar to *Scaphiopus couchii* and *Spea multiplicata*, which were collected in abundance. Therefore it is difficult to understand why *B. cognatus* and *B. speciosus* were not previously detected on the ranch, and it may be possible that both species have increased their abundance since 1948. Additionally, our recent surveys have resulted in the first specimen of *Syrrhophus marnockii* collected in the Sierra Vieja (Owen et al. 2014). *Syrrhophus marnockii* has previously been heard calling in canyons in the Sierra Vieja, but collection attempts have been unsuccessful over the last 60 years (Jameson and Flury 1949; TJL, unpublished data).

Over the 14 years that we have been surveying this site for amphibians and reptiles, only three species that were detected in 1948 have yet to be encountered: *Ambystoma mavortium*, *Lampropeltis alterna*, and *Salvadora grahamiae*. The exact reasons for the failure to detect these species is unknown. *Ambystoma mavortium* was collected in abundance from an artificial, dugout pond surrounded by tobosa-grama association in 1948. Additional voucher specimens of *A. mavortium* exist from 1947 (TNHC 1102: lot of 100 specimens) and 1949 (TNHC 8920) from C. E. Miller Ranch, and most were collected from a series of small ponds surrounding the 1948 locality. Many of these specimens collected in 1947 (TNHC 1102) have reduced external gill size and show the beginnings of adult patterning, indicating that these individuals do undergo metamorphosis to terrestrial adults. The multiple localities were *A. mavortium* were found suggests that a meta-population may have once existed on the ranch and in the Valentine Plain. The landowners have reported seeing this species in ponds and crossing roads as recently as the 1970’s (Miller Family, pers. comm.), and suggest that a series of droughts may have resulted in the local extirpation of this species. Detailed survey work for *A. mavortium* in west Texas is lacking, but this species has recently been detected in the Davis Mountains to the east (iNaturalist 3956528; http://www.inaturalist.org/observations/3956528). It is possible that a series of high rainfall years in succession may allow individuals to disperse back to C. E. Miller Ranch. Failure to detect *L. alterna* is attributed to its cryptic nature. A single *L. alterna* (TNHC 4181) was collected in 1948 from Fox Canyon in the Sierra Vieja and targeted surveys for this species in canyons within the Sierra Vieja have been unsuccessful in locating this species. Unfortunately, we cannot explain our failure to encounter *S. grahamiae* during recent surveys. Seven specimens of *S. grahamiae* (TNHC 3153, 3369, 3563, 3834, 3924, 3993, 4264) were collected in 1948, but extensive diurnal and crepuscular surveys across multiple associations within the Sierra Vieja did not produce a single specimen. We suggest that future surveys should focus on sampling within canyons for *L. alterna* and the plateaus on top of the Sierra Vieja for *S. grahamiae*.

Future surveys may still detect new species from this study site. For example, both the Glossy Snake (*Arizona elegans*) and the Western Massassauga (*Sistrurus tergeminus*) have been found in the Valentine Plain between the Sierra Vieja and the Davis Mountains. The nearest known occurrences of both *A. elegans* (TNHC 95847) and *S. tergeminus* (Sul Ross State University [SRSU] 6616) are ca. 23 km and 28 km respectively (measured from the C. E. Miller Ranch Headquarters), to the east along U.S. Hwy 90. Several other additional species of reptiles can be found ca. 45 km to the east in the Davis Mountains and ca. 18 km to the west along the Rio Grande that have not been detected in the Sierra Vieja and at the study site. Species present in the Davis Mountains that have not been detected in the Sierra Vieja include Greater Short-horned Lizard (*Phrynosoma hernandesi*), North American Racer (*Coluber constrictor*), Milksnake (*Lampropeltis triangulum*), Baird’s Ratsnake (*Pantherophis bairdi*), Trans-Pecos Black-headed Snake (*Tantilla cucullata*), and Copperhead (*Agkistrodon contortrix*). Species present along the Rio Grande but not found in the Sierra Vieja include the Marbled Whiptail (*Aspidoscelis marmorata*), Big Bend Spotted Whiptail (*A. scalaris*), Common Side-blotched Lizard (*Uta stansburiana*), Great Plains Ratsnake (*Pantherophis emoryi*), Spiny Softshell (*Apalone spinifera*), and Mexican Plateau Slider (*Trachemys gaigeae*). At our study site, suitable habitat for some of these species (e.g., *A. spinifera, T. gaigeae*) does not occur, though suitable habitats for many of these other species do appear to be present and match nearby habitats where these species can be found. Instead, the primary constraint for these species is the lack of corridors to connect proximate populations to the Sierra Vieja and the study site. The Valentine Plain between the Sierra Vieja and the Davis Mountains serves as a barrier to impede the movement of montane species and the Sierra Vieja themselves, along with the rim rock on the western edge of the range, serve as a barrier to impede the movement of flatland species between the Rio Grande and the Valentine Plain (York 1949).

The involvement of amateurs and professionals during the 2007 Texas Herpetological Society (THS) spring field meet helped to generate records of previously reported and new species of amphibians and reptiles from C. E. Miller Ranch. Yearly field meets conducted by the THS functionally serve as bioblitzes, rapid assessments of biodiversity for a given area. Specifically, four new species of reptiles were collected during the THS field meet: *Holbrookia maculata*, *Trimorphodon vilkinsonii*, *Rena dissecta*, and *Crotalus lepidus*. Many additional vouchers of previously known species collected during this trip also helped record species from additional habitat associations. While no systematic effort was made to voucher photographs, observations of amphibians and reptiles from the THS field meet, and additional trips to the ranch by various individuals, have been posted to the Herps of Texas project on iNaturalist (https://www.inaturalist.org/projects/herps-of-texas). Although none of these photographic records represented new species occurrences for the property, we recognize the significance of these records in confirming the presence, distribution, and persistence of the C. E. Miller Ranch herpetofauna. The creation of focused taxon- or locality-based projects on repositories such as iNaturalist could be crucial in identifying rarely seen or even new taxa (Deutsch et al. 2017). While we concede there are limitations of photographic records when compared to voucher-based surveys, we encourage the incorporation of citizen science observations with standard voucher-based surveys and collections and further acknowledge the important contributions such photographs can provide to traditional surveys (e.g., Spear et al. 2017).

In sum, our recent surveys from 2004–2017 have been successful in detecting the vast majority of species previously detected from this study site in 1948, though we have been unable to detect three species. While we are unable to determine the exact reasons, we believe that two of these species (*Lampropeltis alterna*, *Salvadora grahamiae*) are likely still present at this site, but their cryptic nature has precluded their detection; the third species, *Ambystoma mavortium*, is presumed to be locally extirpated. Additionally, our recent surveys have been able to detect six species that were previously undetected during the 1948 survey. These six species were likely present at the study site in 1948, but their cryptic nature, low abundance, or unfavorable environmental conditions prevented their detection. Follow-up surveys like ours are important to document changes in species diversity or assemblage through time, even though the exact causal relationships between change in diversity and factors influencing this change (i.e., land management decisions, climate change) remain uncertain. With high levels of amphibian and reptile diversity in the Chihuahuan Desert (Jones et al. 2016), our results highlight the importance of land stewardship and environmentally conscious land management decisions on maintaining the diversity of amphibians and reptiles in the region.

## Appendix 1

Voucher specimens from C. E. Miller Ranch in Jeff Davis and Presidio counties, Texas, USA collected during recent surveys from 2004–2017. Numbers in parentheses indicate the number of specimens collected. TCWC = Biodiversity Research and Teaching Collections, Texas A&M University (formerly Texas Cooperative Wildlife Collections); TNHC = Biodiversity Collections, The University of Texas at Austin (formerly Texas Natural History Collections).

*Bufo cognatus* (5): TNHC 67036, 67037, 67353, 67354, 95841.

*Bufo debilis* (14): TCWC 89827; TNHC 67333–67337, 89580, 89581, 89583, 89887, 95852, 97394, 97481, 99572.

*Bufo punctatus* (15): TNHC 67325–67327, 67375–67377, 89589, 89590, 95850, 97122, 97123, 98914, 99589, 99590, 104419.

*Bufo speciosus* (10): TNHC 67034, 67035, 67381, 68912, 98920, 99580, 104397–104400.

*Syrrhophus marnockii* (1): TNHC 92230.

*Hyla arenicolor* (7): TNHC 67029–67031, 67403, 89446, 95843, 97033.

*Gastrophryne olivacea* (7): TNHC 65363, 65364, 67324, 68907, 97488, 194414, 104415.

*Rana berlandieri* (6): TCWC 88229; TNHC 67032, 67033, 89606, 89607, 104420.

*Scaphiopus couchii* (9): TNHC 67328, 67329, 68908, 89611, 89663, 91983, 95859, 97410, 99574.

*Spea multiplicata* (16): TNHC 67338–67344, 68909–68911, 89445, 89889, 97128, 104416–104418.

*Terrapene ornata* (3): TNHC 92309, 99558, 99559.

*Kinosternon flavescens* (25): TNHC 65365, 65366, 66970–66972, 69375–69378, 89642, 95868, 95869, 97118–97120, 99560–99562, 99583, 99584, 104401–104404, 104450.

*Crotaphytus collaris* (3): TNHC 66883, 66884, 104393.

*Coleonyx brevis* (7): TNHC 66997–67000, 67347, 89591, 99587.

*Cophosaurus texanus* (15): TNHC 67005–67008, 89592, 89593, 98915–98917, 98919, 98921–98924, 98926.

*Holbrookia maculata* (6): TNHC 67018, 68818, 85316, 85317, 89599, 89600.

*Phrynosoma cornutum* (6): TNHC 66889, 68809, 85350, 85351, 89579, 95840.

*Phrynosoma modestum* (4): TNHC 66885, 68817, 89603, 89604.

*Sceloporus cowlesi* (7): TNHC 67014, 67015, 89614–89617, 99592.

*Sceloporus poinsettii* (1): TNHC 100867

*Urosaurus ornatus* (9): TCWC 89836–89838; TNHC 67019–67022, 89621, 89622.

*Aspidoscelis exsanguis* (16): TCWC 88247, 89833–89835, 91583, 92136; TNHC 67009–67013, 67016, 67017, 89584, 89585, 89659.

*Aspidoscelis inornata* (15): TCWC 88238, 88239, 89832; TNHC 67023–67028, 89586, 89587, 89662, 97361, 97393, 104396.

*Aspidoscelis tesselata* (8): TNHC 66886–66888, 89501, 89502, 89588, 89660, 89661.

*Plestiodon obsoletus* (6): TCWC 89841; TNHC 67001, 67002, 85236, 89509, 89623.

*Plestiodon tetragrammus* (4): TCWC 91823; TNHC 67003, 67004, 67348.

*Bogertophis subocularis* (2): TNHC 66576, 99599.

*Diadophis punctatus* (2): TNHC 89595, 97115.

*Gyalopion canum* (1): TNHC 89597.

*Heterodon kennerlyi* (3): TNHC 66582, 89598, 104421.

*Hypsiglena jani* (4): TCWC 91824; TNHC 66598, 66600, 89601.

*Lampropeltis splendida* (4): TNHC 66595, 66596, 89496, 89582.

*Masticophis flagellum* (5): TNHC 66577, 66579, 97125, 97126, 99585.

*Masticophis taeniatus* (1): TNHC 66581.

*Pituophis catenifer* (2): TNHC 66752, 89846.

*Rhinocheilus lecontei* (1): TNHC 89515.

*Salvadora deserticola* (4): TNHC 66714, 66715, 89608, 89609.

*Sonora semiannulata* (4): TNHC 66591–66594.

*Tantilla hobartsmithi* (4): TCWC 89846; TNHC 66741–66743.

*Tantilla nigriceps* (2): TNHC 89679, 89694.

*Thamnophis cyrtopsis* (8): TCWC 92430; TNHC 66716, 66717, 85444, 85445, 85869, 89618, 89619.

*Thamnophis marcianus* (6): TNHC 66590, 85450, 89620, 99579, 104405, 104451.

*Trimorphodon vilkinsonii* (3): TNHC 66487, 66513, 89913.

*Rena dissecta* (2): TNHC 66486, 67346.

*Rena humilis* (1): TNHC 68780.

*Crotalus atrox* (9): TCWC 88252; TNHC 65741, 66497, 66540–66542, 89886, 99576, 104394.

*Crotalus lepidus* (1): TNHC 100146.

*Crotalus ornatus* (2) TNHC 66543, 89706.

*Crotalus scutulatus* (17): TCWC 93138; TNHC 66531–66533, 66537–66539, 66881, 66882, 68735, 68736, 89648, 89782, 89855, 95871, 97398, 97399.

*Crotalus viridis* (2): TNHC 66528, 97121.
