# Corrigenda: Davis DR, LaDuc TJ (2018) Amphibians and reptiles of C. E. Miller Ranch and the Sierra Vieja, Chihuahuan Desert, Texas, USA. ZooKeys 735:97–130. https://doi.org/10.3897/zookeys.735.22200

**DOI:** 10.3897/zookeys.744.25059

**Published:** 2018-03-20

**Authors:** Drew R. Davis, Travis J. LaDuc

**Affiliations:** 1 Department of Biology, University of South Dakota, 414 East Clark Street, Vermillion, South Dakota 57069, USA; 2 Biodiversity Collections, The University of Texas at Austin, 10100 Burnet Road, PRC 176–R4000, Austin, Texas 78758, USA

In our recently published checklist, we mislabeled one of the species of lizards in Figure 6. In error, we listed Figure 6D as a Chihuahuan Spotted Whiptail (*Aspidoscelis exsanguis*) instead of the correct identification as a Little Striped Whiptail (*Aspidoscelis inornata*). Additionally, on 6 February 2018 (ironically, the date this manuscript was published), we received a photographic voucher of an adult Eastern Patch-nosed Snake (*Salvadora grahamiae*) from the Miller family (Jill Miller), taken from the lechuguilla-beargrass association on the top of a mesa in the Sierra Vieja (TNHC 107584). This new voucher reduces the number of species not detected from the historic 1948 survey from three to two.

